# Alternative Splicing Generates Different Parkin Protein Isoforms: Evidences in Human, Rat, and Mouse Brain

**DOI:** 10.1155/2014/690796

**Published:** 2014-07-16

**Authors:** Soraya Scuderi, Valentina La Cognata, Filippo Drago, Sebastiano Cavallaro, Velia D'Agata

**Affiliations:** ^1^Department of Bio-Medical Sciences, Section of Anatomy and Histology, University of Catania, Via S. Sofia, No. 87, 95123 Catania, Italy; ^2^Functional Genomics Center, Institute of Neurological Sciences, Italian National Research Council, Via Paolo Gaifami, No. 18, 95125 Catania, Italy; ^3^Department of Clinical and Molecular Biomedicine, Section of Pharmacology and Biochemistry, University of Catania, Viale Andrea Doria 6, 95125 Catania, Italy

## Abstract

Parkinson protein 2, E3 ubiquitin protein ligase (*PARK2*) gene mutations are the most frequent causes of autosomal recessive early onset Parkinson's disease and juvenile Parkinson disease. Parkin deficiency has also been linked to other human pathologies, for example, sporadic Parkinson disease, Alzheimer disease, autism, and cancer. *PARK2* primary transcript undergoes an extensive alternative splicing, which enhances transcriptomic diversification. To date several *PARK2* splice variants have been identified; however, the expression and distribution of parkin isoforms have not been deeply investigated yet. Here, the currently known *PARK2* gene transcripts and relative predicted encoded proteins in human, rat, and mouse are reviewed. By analyzing the literature, we highlight the existing data showing the presence of multiple parkin isoforms in the brain. Their expression emerges from conflicting results regarding the electrophoretic mobility of the protein, but it is also assumed from discrepant observations on the cellular and tissue distribution of parkin. Although the characterization of each predicted isoforms is complex, since they often diverge only for few amino acids, analysis of their expression patterns in the brain might account for the different pathogenetic effects linked to *PARK2* gene mutations.

## 1. Introduction 

Homozygous or compound heterozygous mutations of Parkinson protein 2, E3 ubiquitin protein ligase (*PARK2*) geneare cause (50% of cases) of autosomal recessive forms of PD, usually without atypical clinical features.* PARK2* mutations also explain ~15% of the sporadic cases with onset before 45 [1, 2] and act as susceptibility alleles for late-onset forms of Parkinson disease (2% of cases) [3]. Along with Parkinsonism forms,* PARK2* gene has been linked to other human pathologies, such as Alzheimer disease [4], autism [5], multiple sclerosis [6], cancer [7, 8], leprosy [9], type 2 diabetes mellitus [10], and myositis [11].

*PARK2* gene is located in the long arm of chromosome 6 (6q25.2-q27) and spans more than 1.38 Mb [12, 13]. From the cloning of the first human cDNA [12, 13],* PARK2 *genomic organization was thought to include only 12 exons encoding one transcript. Many evidences now demonstrate the existence of additional exonic sequences, which can be alternatively included or skipped in mature mRNAs. To date, dozens of* PARK2* splice transcripts have been described [14] and have been demonstrated to be differentially expressed in tissue and cells [15–21]. These multiple* PARK2* splice variants potentially encode for a wide range of distinct protein isoforms with different structures and molecular architectures. However, the characterization and the distribution of these isoforms have not been deeply detailed yet. While studying* PARK2* splice variants mRNAs is relatively simple, differentiating protein isoforms is more complex, since they often diverge only for few amino acids. The complexity of this task could explain the small number of scientific papers on this topic. However, solving this riddle is fundamental to comprehend the precise role of* PARK2* in human diseases. The tissue and cell specific expression pattern of* PARK2 *isoforms, in fact, might account for the different pathogenetic effects linked to this gene.

In this review, we briefly describe the structure of* PARK2* gene, its currently known transcript products, and the predicted encoded protein isoforms expressed in human, rat and mouse; the latter are two commonly used animal models for studying human diseases. Then, we illustrate the expression of these isoforms by recapitulating the major literature evidences already available, which have previously unknowingly demonstrated their existence. We focus on the expression and cellular distribution of parkin isoforms in the brain. Finally, we collect in a panel the different* parkin* antibodies, commercially available, which could be useful for the characterization of the isoforms expression and distribution.

## 2. *PARK2* Alternative Splice Transcripts Produce Isoforms with Different Structures and Functions

**Figure 1:**
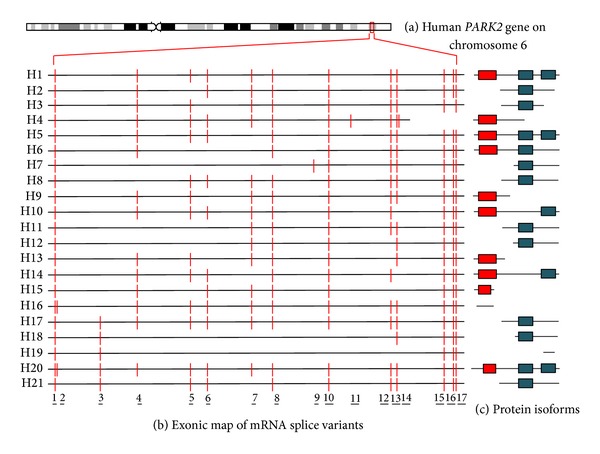
Chromosomal localization, exonic structure of alternative splice variants, and corresponding predicted protein isoforms of human* PARK2*. (a) Cytogenetic location of human* PARK2 *gene (6q26). (b) Exon organization map of the 21 human* PARK2* splice variants currently known. Exons are represented as red bars. The size of introns (black line) is proportional to their length. The codes on left refer to gene identifiers reported in Table 1. (c) Predicted molecular architecture of* PARK2* isoforms. Red boxes represent UBQ domain and blue boxes represent IBR domains.

**Figure 2:**
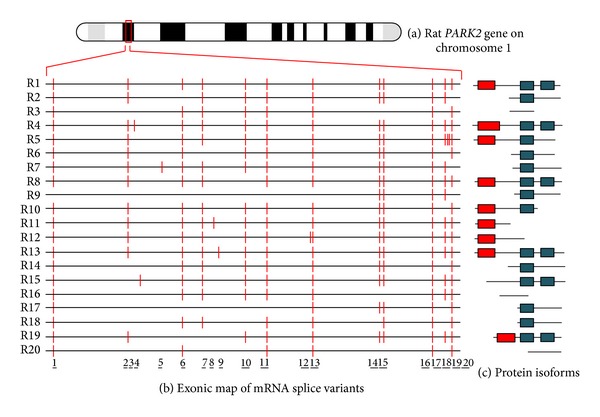
Chromosomal localization, exonic structure of alternative splice variants, and corresponding predicted protein isoforms of rat* PARK2*. (a) Cytogenetic location of rat* PARK2 *gene (1q11). (b) Exon organization map of the 20 rat* PARK2* splice variants currently known. Exons are represented as red bars. The size of introns (black line) is proportional to their length. The codes on left refer to gene identifiers reported in Table 2. (c) Predicted molecular architecture of* PARK2* isoforms. Red boxes represent UBQ domain and blue boxes represent IBR domains.

**Figure 3:**
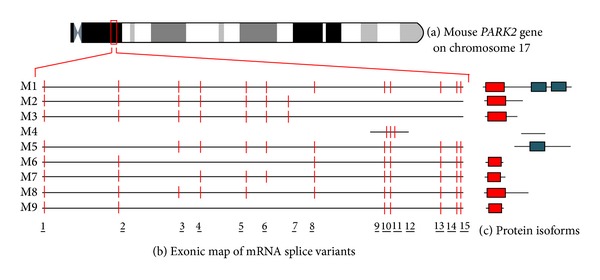
Chromosomal localization, exonic structure of alternative splice variants, and corresponding predicted protein isoforms of mouse* PARK2*. (a) Cytogenetic location of mouse* PARK2 *gene (A3.2-A3.3). (b) Exon organization map of the 9 mouse* PARK2* splice variants currently known. Exons are represented as red bars. The size of introns (black line) is proportional to their length. The codes on left refer to gene identifiers reported in Table 3. (c) Predicted molecular architecture of* PARK2* isoforms. Red boxes represent UBQ domain and blue boxes represent IBR domains.

To date, 26 human different cDNAs, corresponding to 21 unique* PARK2 *alternative splice variants, have been described and are summarized in Figure 1 and Table 1. These mature transcripts are derived from the combination of 17 different exonic regions. Similarly, 20* PARK2* transcripts (20 exons) have been characterized in rat (Figure 2 and Table 2) and 9 (15 exons) in mouse (Figure 3 and Table 3). All of them have been carefully described in our previous paper [14]. For each of these variants, the encoded protein isoform, the corresponding molecular weight, and isoelectric point have been predicted and reported in Tables 1, 2, and 3. H8/H17, H9/H13, and H7/H18 isoforms show the same molecular weight and isoelectric point (Table 1), since they have the same amino acid composition; similarly, R2/R7/R14, R17/R18, and R3/R16 show the same primary structure, as shown in Table 2. Although equal, these proteins are encoded by different splice variants which probably produce the same protein with different efficiency.

In addition to primary structures, molecular architectures and domains composition have also been evaluated (Figures 1, 2, and 3 panels (b) and (c)). As previously described, the original (canonical)* PARK2 *protein (Accession number BAA25751.1) [12] comprises an N-terminal ubiquitin-like (UBQ) domain and two C-terminal in-between ring fingers (IBR) domains. The UBQ domain targets specific protein substrates for proteasome degradation, whereas IBR domains occur between pairs of ring fingers and play a role in protein quality control.* PARK2* encoded isoforms structurally diverge from the canonic one for the presence or absence of the UBQ domain and for one of or both IBR domains. Moreover, when the UBQ domain is present, it often differs in length from that of the canonical sequence. Interestingly, some isoforms miss all of these domains.

The different molecular architectures and domain composition of isoforms might roughly alter also their functions. Parkin protein acts as an E3 ubiquitin ligase and is responsible of substrates recognition for proteasome-mediated degradation.* PARK2* tags various types of proteins, including cytosolic (Synphilin-1, Pael-R, CDCrel-1 and 2a, *α*-synuclein, p22, and Synaptotagmin XI) [25–29], nuclear (Cyclin E) [15], and mitochondrial ones (MFN1 and MFN2, VDAC, TOM70, TOM40 and TOM20, BAK, MIRO1 and MIRO2, and FIS1) [30–34]. The number of targets is so high that parkin protein results involved in numerous molecular pathways (proteasome-degradation, mitochondrial homeostasis, mitophagy, mitochondrial DNA stability, and regulation of cellular cycle). To date it is unknown if all these functions are mediated by a single protein or by different isoforms. However, considering that parkin mRNAs have a different expression and distribution in tissues and cells [14], which should be also mirrored at the protein level, it is reasonable to hypotisize that these distinct isoforms might perfom specific functions and could be differentially expressed in each cellular phenotype. Each* PARK2* splice variants may acts in different manner to suit cell specific needs. This hypothesis is supported by previous evidences showing different and even opposite functions of other splice variants, such as BCl2L12 pattern expression related to cellular phenotype [35]. Finally, based on the extensive alternative splicing process of* PARK2 *gene, we cannot rule out that additional splice variants with different functions (beyond those listed) may exist.

## 3. Evidences of Multiple Parkin Isoforms in Brain

A remarkable number of papers have demonstrated the existence, in human and other species, of different mRNA parkin variants [15–21]. However, few of them have investigated parkin isoforms existence, and some have done it without the awareness of* PARK2* complex splicing [23, 36, 37]. In fact, although many mRNA parkin splice variants have been cloned, the corresponding proteins have been only deduced through the analysis of the longest open reading frame and uploaded on protein databases as predicted sequences. To date many questions are still unanswered: Are all mRNA parkin splice variants translated? Does a different expression pattern of parkin proteins, in tissue and cells, exist? Does each protein isoform have a specific function? In the following paragraphs we try to answer these questions by summarizing the knowledge accumulated over the last three decades on parkin expression and distribution in human, rat, and mouse brain. Existing data are reinterpreted by considering the complexity level of* PARK2* gene splicing described above.

Many conflicting data emerges in the literature regarding the number and relative electrophoretic mobility of parkin proteins. While the majority of papers reported only a band of ~52 kDa corresponding to the canonical parkin isoform, also known as* full length parkin*, additional bands (from ~22 kDa to ~100 kDa) both in rodent [23, 28, 36–41] and human brain regions were also detected [22–25, 39, 42–45].

Parkin was observed both in rat central and peripheral nervous system. Two major bands of ~50 and ~44 kDa were recognized in cell extracts from rat* Substantia Nigra* (SN) and cerebellum by western blot analysis. In adrenal glands there were visualized several immunoreactive bands of 50, 69–66, and 89 kDa [36]. Additional bands were also observed in primary cultures of cortical type I astrocytes [37].

Similar result was observed in mouse brain homogenate: a major band of 50 kDa and fainter bands of ~40 and 85/118 kDa were identified on immunoblot. In all these papers, lower and higher molecular weight bands were described as posttranslational modification or proteolytic cleavage of 52 kDa canonical protein or heterodimers resulting from the interaction of parkin with other proteins [42]. However, we speculate that they might correspond to multiple parkin isoforms with different molecular weight.

In knocked-out mice for parkin exon 2, several unexpected bands were also observed on immunoblot. This was interpreted as antibody cross-reactivity with nonauthentic parkin protein [46]. However, as shown in Figure 3, these bands might represent isoforms encoded by splice variants not containing the deleted exon (i.e., M5 and M4).

Parkin expression was also demonstrated in human brains of normal and sporadic Parkinson disease (PD) subjects, but it was absent in any regions of AR-JP brain [22, 23]. A major band of 52 kDa and a second fainter band of ~41 kDa were observed on immunoblot from human frontal cortex of PD patients and control subjects [22]. Parkin expression was also observed in Lewy bodies (LBs), characteristic neuronal inclusions in PD brain. However, in this regard we highlight widely varying results. Initially, the parkin protein expression was reported in neurons of the SN, locus coeruleus, putamen, and frontal lobe cortex of sporadic PD and control individuals but no parkin-immunoreactivity (IR) was found in SN LBs of PD patients [22, 23]. Later on, parkin-IR was described in nigral LBs of four related human disorders, sporadic PD, *α*-synuclein-linked PD, LB positive parkin-linked PD, and dementia with LBs (DBL) [24]. These discrepant results might be due to the antibodies used. In fact, as shown in Table 4, aligning the epitope sequence recognized by the antibody to each isoform sequence, we discovered that every antibody identifies a pool of different isoforms.

In accord with this hypothesis, we also explain discordant results observed by Schlossmacher et al. (2002) regarding the cellular distribution of the protein. In fact, they described strongly labeled cores of classical intracellular LBs in pigmented neurons of the SN in PD and DLB patients by using HP2A antibody, whereas HP1A and HP7A antibodies intensively labeled cytoplasmic parkin, in a granular pattern, of cell bodies and proximal neurites of dopaminergic neurons in both diseased and normal brains [24]. These results might represent a different cellular expression profile of parkin isoforms in healthy and diseased human brains.

This hypothesis is supported by another study demonstrating a different expression profile of parkin mRNA splice variants in frontal cortex of patients with common dementia with LB, pure form of dementia with LB, and Alzheimer disease suggesting the direct involvement of isoform-expression deregulation in the development of such neurodegenerative disorders [17]. To date there exists only one paper that has dealt with parkin amino acid sequencing [47]. Trying to ensure that the signal observed on human serum by western blot analysis belongs to parkin protein, they cut off the area on the blot between 50 and 55 kDa in two separate pieces and performed a MALDI-TOF analysis on each. Peptides peaks analysis revealed the presence of six other proteins with similar sequence to canonical one. However, authors did not even speculate that they could represent additional parkin isoforms.

Further evidences on the existence of multiple isoforms come from the conflicting data on their tissue and cellular distribution. Parkin protein is particularly abundant in the mammalian brain and retina [22, 23, 36, 48, 49]. In human, parkin immunoreactivity (IR) has been observed in SN, locus coeruleus, putamen, and frontal lobe cortex [22, 23]. Similarly, it has been strongly measured in rat hippocampus, amygdaloid nucleus, endopiriform nucleus, cerebral cortex, colliculus, and SN (pars compacta and pars reticulata) [37, 50].

Analog parkin distribution was reported in mouse. Most immunoreactive cells were found in the hindbrain. In the cerebellum only the cells within the cerebellar nuclei were positive, while the structures located in the mesencephalon presented moderate to strong immunopositivity. In the ventral part of the mesencephalon the red nucleus showed large strongly stained cells. In the SN moderate parkin immunoreactivity was confined to the pars reticulate. In the dorsal mesencephalon, immunopositive cells were found in the intermediate and deep gray layer of the superior colliculus and in all parts of the inferior colliculus [12, 36, 41, 51].

Although in most brain regions good correlations between parkin-IR and mRNA were observed, incongruent data emerged from some paper in rat SNc (*substantia nigra pars compacta*), hippocampus, and cerebellar Purkinje cells distribution, where mRNA was detected but no parkin-IR was revealed [23, 36].

Furthermore, in an early study, parkin was described in cytoplasm, in granular structure, and in neuronal processes but was absent in the nucleus [22]. Subsequently other studies reported also its nuclear localization [23, 37, 48, 52–54]. Finally, some papers have also observed a small mitochondrial pool of the protein [55, 56]. All these evidences have suggested that protein could localize to specific subcellular structure under some circumstances. However, it is also reasonably hypothesized that a specific pattern of subcellular distribution of parkin isoforms is related to each cellular phenotype, since in all these papers, protein immunolocalization was performed by using antibodies recognizing different epitopes. Some discrepancies are also observed in the expression of parkin in the SNc of patients affected by other forms of parkinsonism [23].

Brain isoforms might have different species-specific biochemical characteristics, when comparing murine versus human parkin. In fact, it has been shown that mouse protein is easily extracted from brain by high salt buffer, instead human parkin is only extracted with harsher buffers, especially in elderly. This suggested that human parkin becomes modified or interacts with other molecules with age, and this alters its biochemical properties [42]. However, we cannot rule out that this may correlate to a specific expression pattern of isoforms with different biochemical properties in the brains of rodents and humans relative to age.

All of these observations were also supported by contradictory results emerging from clinical studies. Initially, recessive mutations in the parkin gene were related to sporadic early onset parkinsonism [2]; however, the mode of transmission was subsequently rejected by other genetic studies with not only homozygous or compound heterozygous mutations, but also single heterozygous mutations, affecting only one allele of the gene [2, 57–61]. It has been suggested that haploinsufficiency is a risk factor for disease, but certain mutations are dominant, conferring dominant-negative or toxic gain of functions of parkin protein [61]. However, in light of the evidence outlined above, it is possible that some single heterozygous mutation might affect gene expression by inducing loss of function of some isoforms and gain of function of other.

## 4. The Diversified Panel of Antibodies Commercially Available against* PARK2*

**Figure 4:**
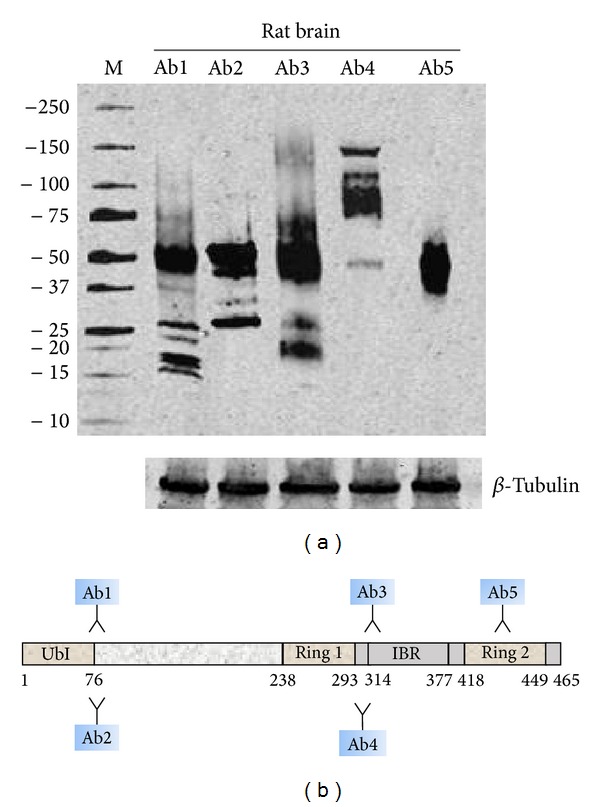
Differential detection of parkin isoforms in rat brain using five anti-parkin antibodies. (a) Representative immunoblot of parkin isoforms in rat brain visualized by using five different antibodies. Ab1, Ab2, Ab3, Ab4, and Ab5 correspond to groups #3, #4, #5, #8, and #9 of Table 5. Immunoblot for *β*-tubulin was used as loading control. (b) Canonical parkin sequence domains recognized by the five antibodies.

To date more than 160* PARK2* antibodies are commercially available. They are obtained from different species (generally rabbit or mouse) and commercialized by various companies.  Table 5 lists 32 commercially available* PARK2* antibodies whose immunogens used are specified by providers in datasheet. Some of them recognize a common epitope, therefore, have been included in the same group. Tables 6, 7, and 8 report, respectively, human, rat, and mouse parkin isoforms recognized by these antibodies. When the amino acid sequence recognized by the antibody perfectly match with the sequence of the protein, it is very likely to get a signal by western blot or immunohistochemistry analysis (this is indicated in the table by “Yes”). Instead, if the antibody recognizes at least 8 consecutive amino acids on the protein, it is likely to visualize a signal both by western blot or immunohistochemistry analysis (this is indicated in the table by “May be”). Finally, if the antibody recognizes less than 8 consecutive amino acids, it could rule out the possibility to visualize a signal on immunoblot or immunohistochemistry analysis (this is indicated in the table by “No”). The use of these 32 antibodies may allow the identification of at least 15 different* PARK2* epitopes (Table 5). Although no epitope is isoform specific, the combinatorial use of antibodies targeting different protein regions may provide a precious aid to decode the exact spectrum of* PARK2* isoforms expressed in tissues and cells. An example of combinatorial use of antibodies has been reported in Figure 4. On rat brain homogenate, these five antibodies raised against different parkin epitopes, revealed the canonical ~50 kDa band, but additional putative bands of higher and lower molecular weight were visualized. This experimental data reinforce the existence of more than one parkin isoform and confirm that the investigation of parkin expression profile should not be restricted to the use of a single antibody. The latter approach, in fact, could not reveal the entire spectrum of parkin variants.

## 5. Conclusion

Alternative splicing is a complex molecular mechanism that increases the functional diversity without the need for gene duplication. Alternative splicing performs a crucial regulatory role by altering the localization, function, and expression level of gene products, often in response to the activities of key signaling pathways [62].* PARK2* gene, as the vast majority of multiexon genes in humans, undergoes alternative splicing [14, 63, 64]. The importance of alternative splicing in the regulation of diverse biological processes is highlighted by the growing list of human diseases associated with known or suspected splicing defects, including PD [65].

Mutations that affect* PARK2* splicing could modify the levels of correctly spliced transcripts, alter their localization, and lead to a loss of function of some of them and/or gain of function of others in time- and cell-specific manner. Even if few, some evidences supporting this hypothesis have been already described. Preliminary studies reported* PARK2* isoforms with defective degradation activity of cyclin E and control of cellular cycle [15] or characterized by altered solubility and intracellular localization [66]. No evidence of gain of function has been reported, but it is plausible, because a functional screen of the* PARK2* splice variants has not been done yet. The huge number of molecular targets attributed to full-size parkin protein could be shared by the others parkin isoforms which could have additional biological activities that until now are uncosidered. In light of this consideration, alteration of the natural splicing of* PARK2* and deregulation in the expression of parkin isoforms might lead to the selective degeneration of dopaminergic neurons in SN of ARJP. However this is a hypothesis, since the functional screen of the* PARK2* splice variants is not available and this field is still unexplored.

All these could, at least in part, justifying the conflicting and heterogeneous data of studies revised in this work, which preceded the knowledge of* PARK2 *alternative splicing and expression of multiple isoforms for this gene. Understanding* PARK2 *alternative splicing could open up new scenarios for the resolution of some Parkinsonian syndrome.

## Figures and Tables

**Table 1:** *Homo sapiens* parkin isoforms.

New code identifier	GI	Protein accession number	aa sequence	Predicted MW	pI
H20	469609976	AGH62057.1	530 aa	58,127	6,41
H1	3063387 121308969 158258616 169790968 125630744	BAA25751.1 BAF43729.1 BAF85279.1 NP_004553.2 ABN46990.1	465 aa	51,65	6,71
H5	284468410 169790970	ADB90270.1 NP_054642.2	437 aa	48,713	7,12
H10	284468412	ADB90271.1	415 aa	46,412	6,91
H14	284516985	ADB91979.1	387 aa	43,485	7,43
H4	34191069	AAH22014.1	387 aa	42,407	8,15
H8	284468407	∗	386 aa	42,52	6,65
H17	284516991	∗	386 aa	42,52	6,65
H21	520845529	AGP25366.1	358 aa	39,592	7,08
H6	169790972	NP_054643.2	316 aa	35,63	6,45
H11	284516981	∗	274 aa	30,615	6,3
H2	20385797	AAM21457.1	270 aa	30,155	6,05
H3	20385801	AAM21459.1	203 aa	22,192	5,68
H12	284516982	∗	172 aa	19,201	6,09
H9	284468408	ADB90269.1	143 aa	15,521	5,54
H13	284516983	ADB91978.1	143 aa	15,521	5,54
H7	194378189	BAG57845.1	139 aa	15,407	6,41
H18	284516993	∗	139 aa	15,393	6,41
H15	284516987	ADB91980.1	95 aa	10,531	8,74
H19	469609974	AGH62056.1	61 aa	6,832	10,09
H16	284516989	ADB91981.1	51 aa	5,348	7,79

H1 represents the canonical sequence cloned by Kitada et al., 1998 [12].

∗ The protein accession number is not present in database.

**Table 2:** *Rattus norvegicus* parkin isoforms.

New code identifier	GI	Protein accession number	aa sequence	Predicted MW	pI
R13	284810438	ADB96019.1	494 aa	54,829	6,46
R4	20385787	AAM21452.1	489 aa	54,417	6,46
R1	7229096 7717034 11464986 11527823 7001383	BAA92431.1 AAF68666.1 NP_064478.1 AAG37013.1 AAF34874.1	465 aa	51,678	6,59
R5	20385789	AAM21453.1	446 aa	49,367	6,59
R8	20385795 284066979	AAM21456.1 ADB77772.1	437 aa	48,734	6,74
R15	520845531	AGP25367.1	421 aa	46,854	6,59
R10	284066981	ADB77773.1	394 aa	43,297	6,06
R19	520845539	AGP25371.1	344 aa	38,558	6,13
R2	18478865	AAL73348.1	274 aa	30,641	6,2
R7	20385793 284810436	AAM21455.1 ADB96018.1	274 aa	30,641	6,2
R14	520845525 520845527	AGP25364.1 AGP25365.1	274 aa	30,669	6,2
R12	284468405	ADB90268.1	256 aa	28,006	6,44
R6	20385791	AAM21454.1	203 aa	22,288	5,42
R11	284468403	ADB90267.1	193 aa	21,253	8,54
R9	20385803	AAM21460.1	177 aa	19,84	5,97
R17	520845535	AGP25369.1	139 aa	15,404	6,29
R18	520845537	AGP25370.1	139 aa	15,404	6,29
R3	18478869	AAL73349.1	111 aa	12,329	6,92
R16	520845533	AGP25368.1	111 aa	12,329	6,92
R20	520845541	AGP25372.1	86 aa	9,929	7,5

**Table 3:** *Mus musculus* parkin isoforms.

New code identifier	GI	Protein accession number	aa sequence	Predicted MW	pI
M1	10179808 118131140 5456929 86577675	AAG13890.1 NP_057903.1 BAA82404.1 AAI13205.1	464 aa	51,617	6,9
M5	220961631	∗	274 aa	30,631	6,54
M2	10179810	AAG13891.1	262 aa	28,7	7,57
M3	10179812	AAG13892.1	255 aa	28,154	8,49
M8	220961637	ACL93283.1	214 aa	23,388	6,51
M7	220961635	ACL93282.1	106 aa	11,482	9,3
M4	74227131	**∗**	75 aa	8,053	8,85
M6	220961633	ACL93281.1	65 aa	7,181	5,62
M9	284829878	ADB99567.1	63 aa	6,967	6,53

*The protein accession number is not present in database.

**Table 4:** Parkin isoforms recognized by antibodies used in some studies.

Name	Target	Recognized Parkin isoforms
M73 (Shimura et al., 1999) [22]	124–137	H1, H4, H5, H8, H9, H10, H13, H14, H17, H20, H21
M74 (Shimura et al., 1999) [22]	293–306	H1, H2, H3, H4, H5, H6, H8, H10, H11, H14, H17, H20, H21
ParkA (Huynh et al., 2000) [23]	96–109	H1, H2, H3, H4, H5, H6, H8, H9, H10, H11, H13, H14, H17, H20, H21
ParkB (Huynh et al., 2000) [23]	440–415	H1, H2, H5, H6, H7, H8, H10, H11, H12, H14, H17, H18, H20, H21
HP6A (Schlossmacher et al., 2002) [24]	6–15	H1, H4, H5, H6, H9, H10, H13, H14, H16, H20
HP7A (Schlossmacher et al., 2002) [24]	51–62	H1, H4, H5, H6, H9, H10, H13, H14, H15, H20
HP1A (Schlossmacher et al., 2002) [24]	84–98	H1, H2, H3, H4, H5, H6, H8, H9, H10, H11, H13, H14, H17, H20, H21
HP2A (Schlossmacher et al., 2002) [24]	342–353	H1, H2, H3, H4, H5, H6, H7, H8, H11, H12, H17, H18, H20, H21
HP5A (Schlossmacher et al., 2002) [24]	453–465	H1, H2, H5, H6, H7, H8, H10, H11, H12, H14, H17, H18, H20, H21

**Table 5:** List of antibodies targeting *PARK2* isoforms.

Antibody group #	Generic name		Target domain
	Trade name	Companies	
#1	H00005071-B01P	Abnova	1 aa–387 aa
	H00005071-D01P	Abnova	
	H00005071-D01	Abnova	

#2	OASA06385	Aviva System biology	83 aa–97 aa
	AHP495	AbD Serotec	
	MD-19-0144	Raybiotech, Inc.	
	DS-PB-01562	Raybiotech, Inc.	
	PAB14022	Abnova	

#3	MCA3315Z	AbD Serotec	288 aa–388 aa
	H00005071-M01	Abnova	

#4	PAB1105	Abnova	62 aa–80 aa
	70R-PR059	Fitzgerald	

#5	PAB0714	Abnova	305 aa–323 aa
	AB5112	Millipore Chemicon	
	R-113-100	Novus biologicals	

#6	P5748	Sigma	298 aa–313 aa
	GTX25667 Parkin antibody CR20121213_GTX25667	GeneTex International Corporation	
	ABIN122870	Antibodies on-line	
	PA1-751	Thermo Fisher Scientific, Inc.	

#7	R-114-100	Novus biologicals	295 aa–311 aa
	Anti-Parkin, aa295-311 h Parkin; C-terminal	Millipore Chemicon	

#8	MAB5512	Millipore Chemicon	399 aa–465 aa
	Anti-Parkin antibody, clone PRK8/05882	Millipore Upstate	
	Parkin (PRK8): sc-32282	Santa Cruz	

#9	Parkin (H-300): sc-30130	Santa Cruz	61 aa–360 aa
	Parkin (D-1): sc-133167	Santa Cruz	
	Parkin (H-8): sc-136989	Santa Cruz	

#10	EB07439	Everest Biotech	394 aa–409 aa
	GTX89242 *PARK2* antibody, internal CR20121213_GTX89242	GeneTex International Corporation	
	NB100-53798	Novus biologicals	

#11	GTX113239 Parkin antibody [N1C1] CR20121213_GTX113239	GeneTex International Corporation	28 aa–258 aa

#12	10R-3061	Fitzgerald	390 aa–406 aa

#13	A01250-40	GenScript	300 aa–350 aa

#14	NB600-1540	Novus biologicals	399 aa–412 aa

#15	ARP43038_P050	Aviva System biology	311 aa–360 aa


Antibodies against canonical *PARK2* isoform (NP_004553.2) were grouped if they recognize the same epitope. To each group was assigned a new identification code (#).

**Table 6:** *Homo sapiens*.

New code identifier	Ab #1	Ab #2	Ab #3	Ab #4	Ab #5	Ab #6	Ab #7	Ab #8	Ab #9	Ab #10	Ab #11	Ab #12	Ab #13	Ab #14	Ab #15
H20	May be (360 aa)	Yes	May be (64 aa)	Yes	Yes	Yes	Yes	Yes	May be (299 aa)	May be (17 aa)	May be (230 aa)	Yes	Yes	Yes	May be (47 aa)
H1	May be (360 aa)	Yes	May be (64 aa)	Yes	Yes	Yes	Yes	Yes	Yes	May be (17 aa)	Yes	Yes	Yes	Yes	May be (47 aa)
H5	May be (333 aa)	Yes	May be (64 aa)	Yes	Yes	Yes	Yes	Yes	May be (271 aa)	May be (17 aa)	May be (202 aa)	Yes	Yes	Yes	May be (47 aa)
H10	May be (311 aa)	Yes	May be (22 aa)	Yes	No	May be (14 aa)	Yes	Yes	May be (250 aa)	May be (17 aa)	May be (230 aa)	Yes	No	Yes	No
H14	May be (283 aa)	Yes	May be (22 aa)	Yes	No	May be (14 aa)	Yes	Yes	May be (222 aa)	May be (17 aa)	yes (partial match 202 aa/231 aa)	Yes	May be (12 aa)	May be (15 aa)	No
H4	Yes	Yes	Yes	Yes	Yes	Yes	Yes	No	May be (299 aa)	No	May be (230 aa)	No	Yes	No	May be (47 aa)
H8	May be (274 aa)	Yes	May be (64 aa)	No	Yes	Yes	Yes	Yes	May be (281 aa)	May be (17 aa)	May be (178 aa)	Yes	Yes	May be (15 aa)	May be (47 aa)
H17	May be (274 aa)	Yes	May be (64 aa)	No	Yes	Yes	Yes	Yes	May be (280 aa)	May be (17 aa)	May be (178 aa)	Yes	Yes	May be (15 aa)	May be (47 aa)
H21	May be (254 aa)	Yes	May be (64 aa)	No	Yes	Yes	Yes	Yes	May be (252 aa)	May be (17 aa)	May be (150 aa)	Yes	Yes	May be (15 aa)	May be (47 aa)
H6	May be (148 aa)	No	May be (64 aa)	No	Yes	Yes	Yes	Yes	Yes	Yes	May be (52 aa)	Yes	Yes	Yes	Yes
H11	May be (162 aa)	No	May be (64 aa)	No	Yes	Yes	Yes	Yes	Yes	Yes	May be (66 aa)	Yes	Yes	Yes	Yes
H2	May be (161 aa)	No	May be (64 aa)	No	Yes	Yes	Yes	Yes	Yes	Yes	May be (67 aa)	Yes	Yes	Yes	Yes
H3	May be (161 aa)	No	May be (64 aa)	No	Yes	Yes	Yes	No	Yes	No	May be (67 aa)	No	Yes	No	Yes
H12	May be (42 aa)	No	May be (42 aa)	No	May be (12 aa)	No	No	Yes	Yes	Yes	No	Yes	May be (39 aa)	Yes	Yes
H9	May be (137 aa)	Yes	No	Yes	No	No	No	No	Yes	No	May be (110 aa)	No	No	No	No
H13	May be (137 aa)	Yes	No	Yes	No	No	No	No	Yes	No	May be (110 aa)	No	No	No	No
H7	May be (27 aa)	No	May be (27 aa)	No	No	No	No	Yes	May be (30 aa)	Yes	No	Yes	May be (24 aa)	Yes	Yes
H18	May be (27 aa)	No	May be (27 aa)	No	No	No	No	Yes	May be (30 aa)	Yes	No	Yes	May be (24 aa)	Yes	Yes
H15	May be (65 aa)	No	No	No	No	No	No	No	No	No	May be (38 aa)	No	No	No	No
H19	No	No	No	No	No	No	No	Yes	No	No	No	No	No	No	No
H16	No	No	No	No	No	No	No	No	No	No	No	No	No	No	No

Yes = perfect match between predicted protein sequence and antibody epitope.

May be = partial match between predicted protein sequence and antibody epitope; in parenthesis number of amino acid matching/total number of amino acid recognized by antibody epitope.

No = matching between predicted protein sequence and antibody epitope is less than 8 consecutive amino acids.

**Table 7:** *Rattus norvegicus*.

New code identifier	Ab #1	Ab #2	Ab #3	Ab #4	Ab #5	Ab #6	Ab #7	Ab #8	Ab #9	Ab #10	Ab #11	Ab #12	Ab #13	Ab #14	Ab #15
R13	May be (306 aa)	May be (5 aa)	May be (69 aa)	May be (14 aa)	Yes	Yes	Yes	May be (66 aa)	May be (248 aa)	May be (14 aa)	May be (180 aa)	May be (15 aa)	May be (48 aa)	May be (13 aa)	May be (48 aa)
R4	May be (307 aa)	May be (5 aa)	May be (69 aa)	May be (14 aa)	Yes	Yes	Yes	May be (66 aa)	May be (247 aa)	May be (14 aa)	May be (179 aa)	May be (15 aa)	May be (48 aa)	May be (13 aa)	May be (49 aa)
R1	May be (307 aa)	May be (5 aa)	May be (70 aa)	May be (14 aa)	Yes	Yes	Yes	May be (66 aa)	May be (249 aa)	May be (14 aa)	May be (180 aa)	May be (15 aa)	May be (49 aa)	May be (13 aa)	May be (49 aa)
R5	May be (305 aa)	May be (5 aa)	May be (69 aa)	May be (14 aa)	Yes	Yes	Yes	May be (31 aa)	May be (247 aa)	May be (14 aa)	May be (179 aa)	May be (15 aa)	May be (48 aa)	May be (13 aa)	May be (48 aa)
R8	May be (279 aa)	May be (5 aa)	May be (69 aa)	May be (14 aa)	Yes	Yes	Yes	May be (66 aa)	May be (221 aa)	May be (14 aa)	May be (153 aa)	May be (15 aa)	May be (48 aa)	May be (13 aa)	May be (48 aa)
R15	May be (254 aa)	May be (5 aa)	May be (73 aa)	May be (14 aa)	Yes	Yes	Yes	May be (66 aa)	May be (248 aa)	May be (14 aa)	May be (153 aa)	May be (15 aa)	May be (49 aa)	May be (13 aa)	May be (49 aa)
R10	May be (173 aa)	May be (5 aa)	May be (69 aa)	May be (14 aa)	Yes	Yes	Yes	May be (9 aa)	May be (248 aa)	No	May be (180 aa)	No	May be (48 aa)	No	May be (48 aa)
R19	May be (162 aa)	No	May be (70 aa)	No	Yes	Yes	Yes	May be (68 aa)	May be (173 aa)	May be (14 aa)	May be (74 aa)	May be (15 aa)	May be (49 aa)	May be (13 aa)	May be (49 aa)
R2	May be (147 aa)	No	May be (72 aa)	No	Yes	Yes	Yes	May be (68 aa)	May be (156 aa)	May be (14 aa)	May be (55 aa)	May be (15 aa)	May be (48 aa)	May be (13 aa)	May be (48 aa)
R7	May be (147 aa)	No	May be (72 aa)	No	Yes	Yes	Yes	May be (68 aa)	May be (153 aa)	May be (14 aa)	May be (55 aa)	May be (15 aa)	May be (48 aa)	May be (13 aa)	May be (49 aa)
R14	May be (149 aa)	No	May be (73 aa)	No	Yes	Yes	Yes	May be (68 aa)	May be (155 aa)	May be (14 aa)	May be (56 aa)	May be (15 aa)	May be (49 aa)	May be (13 aa)	May be (49 aa)
R12	May be (196 aa)	May be (5 aa)	No	May be (14 aa)	No	No	No	May be (9 aa)	May be (138 aa)	No	May be (168 aa)	No	No	No	No
R6	May be (147 aa)	No	May be (69 aa)	No	Yes	Yes	Yes	No	May be (153 aa)	No	May be (55 aa)	No	May be (48 aa)	No	May be (48 aa)
R11	May be (139 aa)	May be (5 aa)	No	May be (14 aa)	No	No	No	No	May be (82 aa)	No	May be (112 aa)	No	No	No	No
R9	May be (60 aa)	No	May be (68 aa)	No	Yes	Yes	Yes	May be (68 aa)	May be (67 aa)	May be (14 aa)	No	May be (15 aa)	May be (48 aa)	May be (13 aa)	May be (48 aa)
R17	May be (25 aa)	No	May be (33 aa)	No	No	No	No	May be (68 aa)	May be (32 aa)	May be (14 aa)	No	May be (15 aa)	May be (22 aa)	May be (13 aa)	May be (35 aa)
R18	May be (25 aa)	No	May be (33 aa)	No	No	No	No	May be (68 aa)	May be (32 aa)	May be (14 aa)	No	May be (15 aa)	May be (22 aa)	May be (13 aa)	May be (35 aa)
R3	May be (87 aa)	No	No	No	No	No	No	May be (8 aa)	May be (86 aa)	No	May be (55 aa)	No	No	No	No
R16	May be (87 aa)	No	No	No	No	No	No	May be (8 aa)	May be (86 aa)	No	May be (55 aa)	No	No	No	No
R20	No	No	No	No	No	No	No	May be (66 aa)	No	May be (14 aa)	No	May be (15 aa)	No	May be (13 aa)	No

Yes = perfect match between predicted protein sequence and antibody epitope.

May be = partial match between predicted protein sequence and antibody epitope; in parenthesis number of amino acid matching/total number of amino acid recognized by antibody epitope.

No = matching between predicted protein sequence and antibody epitope is less than 8 consecutive amino acids.

**Table 8:** *Mus musculus*.

New code identifier	Ab #1	Ab #2	Ab #3	Ab #4	Ab #5	Ab #6	Ab #7	Ab #8	Ab #9	Ab #10	Ab #11	Ab #12	Ab #13	Ab #14	Ab #15
M1	May be (294 aa)	No	May be (61 aa)	May be (13 aa)	May be (18 aa)	Yes	Yes	May be (70 aa)	May be (244 aa)	No	May be (176 aa)	May be (15 aa)	May be (48 aa)	May be (14 aa)	Yes
M5	May be (147 aa)	No	May be (62 aa)	No	May be (18 aa)	Yes	Yes	May be (70 aa)	May be (153 aa)	No	May be (55 aa)	May be (15 aa)	May be (48 aa)	May be (14 aa)	Yes
M2	May be (191 aa)	No	No	May be (13 aa)	No	No	No	No	May be (134 aa)	No	May be (164 aa)	No	No	No	No
M3	May be (192 aa)	No	No	May be (13 aa)	No	No	No	No	May be (135 aa)	No	May be (165 aa)	No	No	No	No
M8	May be (161 aa)	No	No	May be (13 aa)	No	No	No	No	May be (106 aa)	No	May be (136 aa)	No	No	No	No
M7	May be (53 aa)	No	No	No	No	No	No	No	No	No	May be (27 aa)	No	No	No	No
M4	No	No	No	No	No	No	No	No	No	No	No	No	No	No	No
M6	May be (53 aa)	No	No	No	No	No	No	No	No	No	May be (27 aa)	No	No	No	No
M9	May be (53 aa)	No	No	No	No	No	No	No	No	No	May be (27 aa)	No	No	No	No

Yes = perfect match between predicted protein sequence and antibody epitope.

May be = partial match between predicted protein sequence and antibody epitope; in parenthesis number of amino acid matching/total number of amino acid recognized by antibody epitope.

No = matching between predicted protein sequence and antibody epitope is less than 8 consecutive amino acids.
